# Supplementary material for: Effect of the subjective intensity of fatigue and interoception on perceptual regulation and performance during sustained physical activity
Source: PLoS One. 2022 Jan 5;17(1):e0262303. doi: 10.1371/journal.pone.0262303 (PMC8730470; doi:10.1371/journal.pone.0262303)
Supplement: S1 Table — Data presented as mean ± SD. (DOCX) [file pone.0262303.s001.docx]

**S1 Table.** Maximum voluntary contraction (MVC) force for the (dominant) knee extensors, the dominant and non-dominant handgrip (HG) at the beginning of each experimental session.

|  |  |  |  |  | *ANOVA* | | |
| --- | --- | --- | --- | --- | --- | --- | --- |
|  | CON | MOD | SEV |  | *F* | *p* | *η_p_^2^* |
| *KE* | 642.5 ± 164.6 | 648.0 ± 159.3 | 632.7 ± 168.2 |  | 1.80 | 0.174 | 0.058 |
| *Dominant HG* | 465.8 ± 94.0 | 470.1 ± 84.3 | 469.8 ± 87.5 |  | 0.27 | 0.762 | 0.009 |
| *Non-dominant HG* | 445.3 ± 102.5 | 448.3 ± 97.1 | 443.1 ± 100.8 |  | 0.52 | 0.595 | 0.018 |

Data presented as mean ± SD.
