# Supplementary material for: Effect of the subjective intensity of fatigue and interoception on perceptual regulation and performance during sustained physical activity
Source: PLoS One. 2022 Jan 5;17(1):e0262303. doi: 10.1371/journal.pone.0262303 (PMC8730470; doi:10.1371/journal.pone.0262303)
Supplement: S2 Table — Data presented as mean ± SD with Mdn and IQR presented in parentheses. DASS: Depression, Anxiety and Stress Scale. KSS: Karolinska Sleepiness Scale. (DOCX) [file pone.0262303.s002.docx]

**S2 Table.** Emotional states and perceived sleepiness between conditions

|  |  |  |  |  | Friedman’s ANOVA | |
| --- | --- | --- | --- | --- | --- | --- |
|  | CON | MOD | SEV |  | *χ^2^* | *p* |
|  |  |  |  |  |  |  |
| **DASS (A.U.)** |  |  |  |  |  |  |
| *Depression* | 1.73 ± 2.45  (0.0, 1.0) | 2.33 ± 3.28  (2.0, 3.5) | 1.93 ± 2.95  (0.0, 1.0) |  | 2.50 | 0.287 |
| *Anxiety* | 1.93 ± 3.17  (0.0, 1.0) | 1.93 ± 2.85  (0.0, 1.0) | 2.60 ± 4.76  (0.0, 4.0) |  | 1.16 | 0.559 |
| *Stress* | 4.60 ± 5.20  (4.0, 6.0) | 5.00 ± 6.34  (3.0, 8.0) | 4.60 ± 5.49  (4.0, 7.5) |  | 0.63 | 0.732 |
|  |  |  |  |  |  |  |
| **KSS (A.U.)** |  |  |  |  |  |  |
| *Pre HG task* | 2.80 ± 1.79  (2.50, 2.75) | 2.77 ± 1.45  (3.0, 3.0) | 2.97 ± 1.73  (3.0, 2.75) |  | 0.74 | 0.691 |
| *Post HG task* | 2.67 ± 1.67  (2.0, 2.75) | 2.67 ± 1.37  (3.0, 2.50) | 2.70 ± 1.68  (2.5, 3.0) |  | 0.03 | 0.987 |
| *Post KE task* | 2.47 ± 1.43  (2.0, 2.0) | 2.80 ± 1.45  (3.0, 1.75) | 2.97 ± 1.77  (3.0, 2.75) |  | 6.02 | 0.049 |

Data presented as mean ± *SD* with *Mdn* and *IQR* presented in parentheses. DASS: Depression, Anxiety and Stress Scale. KSS: Karolinska Sleepiness Scale
