# Supplementary material for: Effect of the subjective intensity of fatigue and interoception on perceptual regulation and performance during sustained physical activity
Source: PLoS One. 2022 Jan 5;17(1):e0262303. doi: 10.1371/journal.pone.0262303 (PMC8730470; doi:10.1371/journal.pone.0262303)
Supplement: S3 Table — Contrast 1 represents the contrast between CON and the combined experimental manipulations (MOD + SEV). Contrast 2 represents the contrast between MOD and SEV. σ2: residual variance, ICC: intraclass correlation coefficient, AIC: Akaike information criterion, R2 marginal: variance explained by the fixed effects over the total (expected) variance of the dependent variable, R2 conditional: variance explained by the fixed and random effects over the total (expected) variance of the dependent variable, CON: control; MOD: moderate RoF; SEV: severe RoF. Full fixed and random effects of models not presented for clarity. (DOCX) [file pone.0262303.s003.docx]

**S3 Table.** Estimated fixed effects from linear mixed analysis of perceived effort and affect during the KE endurance task.

|  |  | Effort | | | | |  | Affect | | | | |
| --- | --- | --- | --- | --- | --- | --- | --- | --- | --- | --- | --- | --- |
|  |  |  | 95% CI | |  |  |  |  | 95% CI | |  |  |
| **Fixed Effects** | **Contrast** | Estimates | Lower | Upper | *t* | *p* |  | Estimates | Lower | Upper | *t* | *p* |
| Intercept |  | 7.86 | 7.39 | 8.32 | 32.96 | <0.001 |  | -1.84 | -2.44 | -1.23 | -5.96 | <0.001 |
| Condition | *Contrast 1* | -0.90 | -1.08 | -0.72 | -9.83 | <0.001 |  | 0.66 | 0.45 | 0.87 | 6.17 | <0.001 |
|  | *Contrast 2* | -0.06 | -0.30 | 0.18 | -0.51 | 0.612 |  | 0.17 | -0.13 | 0.48 | 1.12 | 0.262 |
| Time | *Linear* | 7.48 | 7.11 | 7.85 | 39.22 | <0.001 |  | -5.90 | -6.84 | -4.96 | -12.26 | <0.001 |
|  | *Quadratic* | -1.21 | -1.55 | -0.86 | -6.84 | <0.001 |  | 0.94 | 0.31 | 1.58 | 2.92 | 0.006 |
| Interaction | *Contrast 1*Time*  *(Linear)* | -0.51 | -1.18 | 0.16 | -1.50 | 0.134 |  | 0.68 | -0.13 | 1.50 | 1.64 | 0.102 |
|  | *Contrast 2*Time*  *(Linear)* | -0.47 | -1.41 | 0.47 | -0.98 | 0.329 |  | -0.84 | -2.11 | 0.42 | -1.30 | 0.194 |
|  | *Contrast 1*Time (Quadratic)* | 0.07 | -0.58 | 0.72 | 0.21 | 0.831 |  | -0.06 | -0.86 | 0.75 | -0.14 | 0.891 |
|  | *Contrast 2*Time (Quadratic)* | 0.14 | -0.77 | 1.06 | 0.30 | 0.765 |  | -0.99 | -2.25 | 0.27 | -1.54 | 0.126 |
| **Random Effects** |  | |  |  |  |  |  |  |  |  |  |  |
|  | σ^2^ | 0.78 | | | | |  | 0.86 | | | | |
|  | ICC | 0.68 | | | | |  | 0.75 | | | | |
| **Model Fit** |  |  |  |  |  |  |  |  |  |  |  |  |
|  | AIC | 1739.700 | | | | |  | 1910.484 | | | | |
|  | R^2^ marginal | 0.67 | | | | |  | 0.39 | | | | |
|  | R^2^ conditional | 0.89 | | | | |  | 0.88 | | | | |

Contrast 1 represents the contrast between CON and the combined experimental manipulations (MOD + SEV). Contrast 2 represents the contrast between MOD and SEV. σ2: residual variance, ICC: intraclass correlation coefficient, AIC: Akaike information criterion, R2 marginal: variance explained by the fixed effects over the total (expected) variance of the dependent variable, R2 conditional: variance explained by the fixed and random effects over the total (expected) variance of the dependent variable, CON: control; MOD: moderate RoF; SEV: severe RoF. Full fixed and random effects of models not presented for clarity.
