# Supplementary material for: Effect of the subjective intensity of fatigue and interoception on perceptual regulation and performance during sustained physical activity
Source: PLoS One. 2022 Jan 5;17(1):e0262303. doi: 10.1371/journal.pone.0262303 (PMC8730470; doi:10.1371/journal.pone.0262303)
Supplement: S4 Table — Contrast 1 represents the contrast between CON and the combined experimental manipulations (MOD + SEV). Contrast 2 represents the contrast between MOD and SEV. σ2: residual variance, τ00participant: individual variance, ICC(participant): intraclass correlation coefficient, SE: standard error, R2 marginal: variance explained by the fixed effects over the total (expected) variance of the dependent variable, R2 conditional: variance explained by the fixed and random effects over the total (expected) variance of the dependent variable. Full fixed effects not presented for clarity. (DOCX) [file pone.0262303.s004.docx]

**S4 Table.** Estimated fixed and random effects from robust linear mixed analysis of the relative (%) RMS amplitude in the vastus lateralis (VL), vastus medialis (VM) and rectus femoris (RF) during the KE endurance task.

|  |  | VL | | |  | VM | | |  | RF | | |
| --- | --- | --- | --- | --- | --- | --- | --- | --- | --- | --- | --- | --- |
| **Fixed Effects** | **Contrast** | *Estimate* | *SE* | *t* |  | *Estimate* | *SE* | *t* |  | *Estimate* | *SE* | *t* |
| Intercept |  | 26.48 | 0.89 | 29.85 |  | 27.13 | 1.36 | 19.95 |  | 22.25 | 1.13 | 19.75 |
| Condition | *Contrast 1* | -1.45 | 0.36 | -4.06 |  | -2.17 | 0.49 | -4.41 |  | 1.03 | 0.45 | 2.29 |
|  | *Contrast 2* | -1.82 | 0.48 | -3.75 |  | -2.94 | 0.66 | -4.47 |  | 0.29 | 0.61 | 0.48 |
| Time | *Linear* | 28.53 | 1.11 | 25.79 |  | 29.48 | 1.53 | 19.32 |  | 2.38 | 1.40 | 18.16 |
|  | *Quadratic* | 2.01 | 1.07 | 1.88 |  | 2.55 | 1.46 | 1.74 |  | 7.16 | 1.35 | 5.30 |
| Interaction | *Contrast 1*Time (Linear)* | -6.22 | 1.97 | -3.16 |  | -5.78 | 2.76 | -2.09 |  | 0.33 | 2.49 | 0.13 |
|  | *Contrast 2*Time (Linear)* | -1.52 | 2.82 | -0.54 |  | -10.23 | 3.82 | -2.68 |  | -0.85 | 3.57 | -0.24 |
|  | *Contrast 1*Time (Quadratic)* | -1.52 | 1.97 | -0.77 |  | -3.87 | 2.77 | -1.40 |  | -1.21 | 2.50 | -0.48 |
|  | *Contrast 2*Time (Quadratic)* | 7.82 | 2.88 | 2.72 |  | 0.89 | 3.88 | 0.23 |  | 2.59 | 3.65 | 0.71 |
| **Random Effect** | *Participant*  *(Intercept)* |  |  |  |  |  |  |  |  |  |  |  |
|  | σ^2^ | 17.80 | | |  | 32.17 | | |  | 28.71 | | |
|  | τ_00participant_ | 17.75 | | |  | 42.17 | | |  | 28.67 | | |
|  | ICC | 0.50 | | |  | 0.57 | | |  | 0.50 | | |
| **Model Fit** |  |  |  |  |  |  |  |  |  |  |  |  |
|  | R^2^ marginal | 0.41 | | |  | 0.26 | | |  | 0.23 | | |
|  | R^2^ conditional | 0.71 | | |  | 0.68 | | |  | 0.62 | | |

Contrast 1 represents the contrast between CON and the combined experimental manipulations (MOD + SEV). Contrast 2 represents the contrast between MOD and SEV. σ^2^: residual variance, τ_00participant_: individual variance, ICC_(participant)_: intraclass correlation coefficient, *SE*: standard error, R^2^ marginal: variance explained by the fixed effects over the total (expected) variance of the dependent variable, R^2^ conditional: variance explained by the fixed and random effects over the total (expected) variance of the dependent variable. Full fixed effects of models not presented for clarity.
