# Supplementary material for: Effect of the subjective intensity of fatigue and interoception on perceptual regulation and performance during sustained physical activity
Source: PLoS One. 2022 Jan 5;17(1):e0262303. doi: 10.1371/journal.pone.0262303 (PMC8730470; doi:10.1371/journal.pone.0262303)
Supplement: Attachment — Submitted filename: Comments #1.docx [file pone.0262303.s005.docx]

This article investigated whether subjective intensity of perceived fatigue, pre-induced through prior upper body activity, differentially impacted performance and altered perceptual (effort) and affective responses during a sustained, isometric contraction in lower body. The authors also explored whether interoceptive awareness moderated the relationship between the perception of fatigue and effort.

I want to congratulate the author for the quality of this very rigorous and well-written scientific paper. This article brings interesting data on how perceived fatigue and effort perception could interact and influence endurance performance. I was naïve on the interoceptive awareness concept, and I think that this strengthen the interest of this paper. I only have some minor comments (and sometimes some personal observations that do not require any answer).

**Introduction :**

Lines 15-16 : perhaps providing a definition of what perception of effort is should strengthen what you say in the first paragraph, i.e. perception of fatigue should be distinguished from perception of effort. I think that in the definition of “perception of fatigue” it should be stated that this state is independent of any previous physical activity.

Line 20: maybe a clear definition of what “self-efficacy” is will make this part clearer for naïve readers

Line 35: give some example when talking about changes in regulatory responses and performance.

Line 54: give example of interoceptive signals

**Material and methods:**

The quality of the figures is very low, especially the one of the figure 1 (i.e. the experimental design). It is impossible to correctly read this figure, please improve the quality

Line 156: it is not clear for me; ROF was assessed 1 min after the beginning of the endurance exercise, and at the end. That’s it? Or was it also recorded every 30sec from the 1^st^ min of the exercise?

Maybe change the subtitle “rating of fatigue (RoF)” for “perception of fatigue”. It will be clearer, since the next subsection is called “perception of effort”.

**Results:**

Table 1: what does “A.U.contraction-1” means?

Lines 297 and 299: what are Mdn ? Median I guess. Maybe the mean value of the ROF could be helpful to see how far it was from the 6 values for the MOD condition

The quality of fig 2 to fig 6 is better than the one of fig 1 but could be much more improved

Paragraph 3.3. It could be a good idea to give the exact time (in sec) for KE endurance performance. We can guess it by checking the fig 2, but it worth it to give the exact values in the text

The results section is already long, I am not sure that table 2 is really needed. Maybe it could better fit in the supp data

Figure 5 should have panel, and you need to use these panels in the text. Further, the legend of one of the panel of the fig 5 is written with a very small police, this is illegible.

Title of the part 3.6: interoceptive and not interceptive

**Discussion:**

Lines 439-442: I guess that this sentence could go in the conclusion rather than in the first paragraph of the discussion

Line 450: while I agree that it was not the aim of this study, it could be good to give some examples for each causes so as to have a general overview of the mechanisms that account for the deleterious effect of prior physical activity on endurance performance in a remote muscle group

Lines 466-473: a personal comment, that is a very interesting paragraph

Line 494: “a new task”; you mean the transfer from handgrip exercise to KE exercise?

Line 515: which ones?

Lines 520-525: personal comment: another experimental session with the HG exercise until failure (or until a maximal RoF score) could have been interested to meet with what you performed in your previous study (ref 25).

Line 573: is there any possibility for people with “low” individual interoception awareness to improve/train this parameter? Because this interoceptive state can moderate the influence of the perception of fatigue in the perception of effort (thus on the endurance performance), it could be useful to give some recommandations on how a subject with low individual interoception awareness could become a subject with high interoception awareness. I am naïve with this concept, so maybe my comment does not make any sense.
